# Varietal and Geographical Discrimination of Greek Monovarietal Extra Virgin Olive Oils Based on Squalene, Tocopherol, and Fatty Acid Composition

**DOI:** 10.3390/molecules25173818

**Published:** 2020-08-21

**Authors:** Theano Mikrou, Elisavet Pantelidou, Niki Parasyri, Andreas Papaioannou, Maria Kapsokefalou, Chrysavgi Gardeli, Athanasios Mallouchos

**Affiliations:** 1 Laboratory of Food Chemistry and Analysis, Department of Food Science and Human Nutrition, Agricultural University of Athens, Iera Odos 75, 118 55 Athens, Greece; thmikrou@aua.gr (T.M.); pantelidouelisavet@gmail.com (E.P.); nikiparasiri96@gmail.com (N.P.); kapsok@aua.gr (M.K.); agardeli@aua.gr (C.G.); 2 Food Analytical & Research Laboratories of Athens, Hellenic Food Authority (EFET), 115 26 Athina, Greece; apapaioannou@efet.gr

**Keywords:** extra virgin olive oil, classification, geographical origin, olive cultivars, squalene, tocopherols, fatty acids, multivariate analysis

## Abstract

Extra virgin olive oil (EVOO) is an important component of the Mediterranean diet and a highly priced product. Despite the strict legislation to protect it from fraudulent practices, there is an increasing demand to characterize EVOOs and evaluate their authenticity. For this purpose, 68 monovarietal EVOOs, originating from three regions of Greece (Peloponnese, Crete, and Lesvos) and two local cultivars (Koroneiki and Kolovi), were obtained during the harvesting period of 2018–2019. Fatty acids, squalene, and tocopherols were determined chromatographically according to official methods in order to study the effect of cultivar and geographical origin. Squalene and γ-tocopherol differed significantly amongst the cultivars tested. Koroneiki samples exhibited higher squalene content than Kolovi samples, whereas the opposite was observed for γ-tocopherol. The tocopherol level was highly geographical dependent, with EVOOs from Peloponnese displaying the highest concentration of α-tocopherol, whereas the content of γ-tocopherol was significantly higher in samples from Lesvos. Unsupervised and supervised multivariate analysis resulted in a satisfactory grouping of EVOOs according to cultivar. γ-Tocopherol, squalene, and the majority of fatty acids were the most discriminant variables, with γ-tocopherol, linoleic, linolenic, and gadoleic acid being present at higher levels in samples from the Kolovi cultivar. Koroneiki samples were characterized with higher levels of squalene, palmitic, palmitoleic, and arachidic acid.

## 1. Introduction

Olive oil consumption has been associated with longevity due to a lower risk of cardiovascular diseases and other diseases such as diabetes, hypertension, obesity, cancer, neurodegenerative diseases, and depression [1,2]. This is attributed to its high content of bioactive ingredients such as squalene, tocopherols, sterols, triterpenic compounds, and polar phenol compounds [3], as well as its high ratio of monounsaturated to saturated fatty acids [4].

Squalene accounts for 90% of the hydrocarbon fraction in olive oils. It is a linear triterpenic polymer of isoprene and an intermediate compound in the biosynthesis of sterols. There is controversial evidence that squalene plays a rather confined role in olive oil stability even at low temperatures [5]. Diverse chromatographic techniques have been used to determine squalene content such as High-Performance Liquid Chromatography (HPLC) [6,7,8] and Gas Chromatography (GC) [9,10,11] after its extraction with the use of organic solvents.

Tocopherols and tocotrienols are lipophilic compounds comprised by a chromanol ring and a hydrophobic side chain with appreciated antioxidant and vitamin E activity [12]. The alpha-homologue comprises 90–95% of the total tocopherol content where the other homologues have been reported in lower amounts [3]. Liquid chromatography, usually combined with fluorescence detection, is the method of choice for the determination of tocols in oils [13].

Extra virgin olive oil (EVOO) is the main source of fat of the Mediterranean diet and is associated with multiple health benefits. Greece ranks third, after Spain and Italy, in olive oil production, accounting for 12.1% of world production, whereas the olive tree cultivation represents ca. 23% of the cultivated area in Greece [14]. More than 90% of the acreage in the country is cultivated with 20 cultivars, which are adapted to a wide range of environmental conditions [15]. ‘Koroneiki’ originating from the southern Peloponnese, and specifically from Koroni of Messinia, is the most dominant cultivar and is cultivated in several geographical regions all over the country. The most important olive oil-producing areas in Greece are the Peloponnese (located in southern Greece), Crete island (located in southern Aegean Sea), and Lesvos island (located in northeastern Aegean Sea).

The genetic characteristics of each variety in combination with a large number of factors, such as climatic conditions, agricultural practices, stage of maturity, harvest period, extraction, and processing technology are closely related to olive oil quality and its chemical composition [3,14], which in turn determine the unique characteristics of premium products with geographical indications (Protected Designation of Origin, PDO; Protected Geographical Indication, PGI) and monovarietal certifications. Hence, numerous attempts have been made to find suitable analytical approaches to differentiate EVOOs according to their cultivar and geographical origin [16]. More specifically, fatty acid composition [17,18,19] and triacylglycerol content [20,21] have been proven useful factors in order to distinguish the different geographical origins and botanical varieties of EVOOs. In addition, sterol, phenol, and terpenoid content [20,22] have been found to differ according to the location of origin, crop year, and phase of maturation. As far as squalene and tocopherols are concerned, their variability can be explained by the genetic factor, and in the case of tocol content, by the geographical origin [7,10,23,24]. Moreover, volatile compound analysis [14,25,26] has provided a satisfactory classification of EVOOs according to geographical origin and cultivar. Currently, the research efforts have focused on the use of metabolomic approaches combined with multivariate statistical analysis [27,28,29,30]. Regardless of the analytical strategy followed, there is a definite need for further research because the chemical composition of olive oil is considerably diverse and complex. 

The objective of the present work was to investigate the effect of cultivar and geographical origin on the squalene and tocopherol content as well as the fatty acid composition of Greek monovarietal EVOOs. For this purpose, samples of the two most prominent local cultivars (Koroneiki and Kolovi) were selected. They originated from the three major olive oil-producing areas of Greece: Crete island, Lesvos island, and Peloponnese (Messenia). Particularly, the Cretan olive oil samples were produced in the four sub-regions (prefectures), namely Chania, Rethymno, Heraklion, and Lasithi, covering the whole island from west to east. Multivariate data analysis was used as an aid for EVOO traceability. The information obtained here will be compiled with other data as a part of a national project to build a Greek olive oil map.

## 2. Results

### 2.1. Squalene and Tocopherol Content

Table 1 presents the genetic and regional variability for squalene and tocopherols content of the extra virgin olive oils studied. Squalene content ranged between 4534 mg/kg (Kolovi cultivar from Lesvos) and 11,919 mg/kg (Koroneiki cultivar from Rethymno), showing a mean squalene concentration of 8161 mg/kg for the whole set of EVOOs analyzed. When ANOVA was performed, significant differences were found between the two varieties. Koroneiki olive oils were characterized by a higher squalene content than those of the Kolovi cultivar (Table 1). Τhe content of squalene in olive oils of the same cultivar (Koroneiki) produced in Crete, Lesvos, and Peloponesse was found to be similar (*p* > 0.05). The lowest and the highest content of squalene was observed in the samples from Lasithi and Rethymno, respectively.

**Figure 1:**
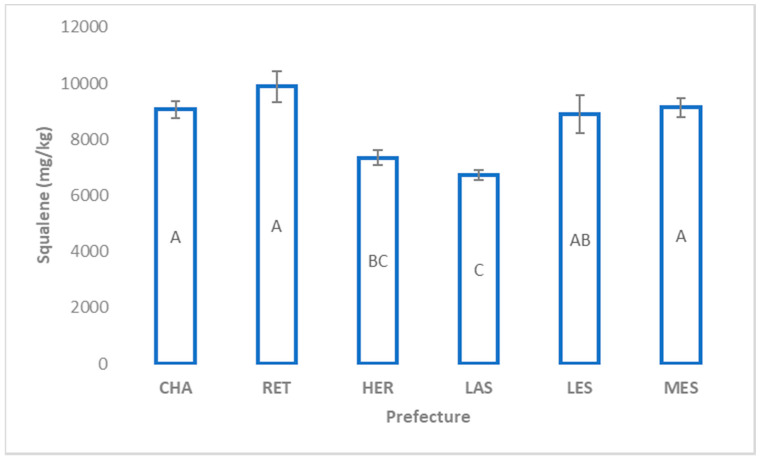
Content of squalene (mean ± standard error) in extra virgin olive oils (EVOOs) from the Koroneiki cultivar produced in various regions of Greece. Different letters indicate significant differences (Tukey’s HSD test, *p* ≤ 0.05) between the prefectures. CHA: Chania; HER: Heraklion; LAS: Lasithi; RET: Rethymno; LES: Lesvos; MES: Messenia.

Figure 1 presents the squalene content of olive oil samples from the four sub-regions (prefectures) of Crete together with those from Messenia and Lesvos. It is observed that the squalene content of olive oil samples from the eastern prefectures (Lasithi and Heraklion) was significantly (*p* ≤ 0.05) lower than that from the western prefectures (Chania and Rethymno) of Crete together with those from Messenia and Lesvos. Differences among sub-regions of the same area and of the same cultivar reveal the possibility that additionally to genetic factors, ecosystems or agricultural practices might influence the level of squalene.

α-Tocopherol was the dominant homologue in EVOOs, representing 94.6% of the total tocol content with a mean value of 116 mg/kg. Its concentration ranged from 79 to 151 mg/kg for samples produced in Chania (western prefecture of Crete) and Messenia (southeastern part of the Peloponnese), respectively. As shown in Table 1, its concentration did not differ significantly between the two olive oil cultivars studied (*p* > 0.05). On the other hand, γ-tocopherol was found at least 10-fold less than α-tocopherol content, but it seems that its concentration is cultivar-dependent (Table 1). EVOOs from the Kolovi cultivar had approximately three times higher γ-tocopherol content than those from the Koroneiki cultivar.

Among olive oil samples of the same variety (Koroneiki) produced from different regions, the α- and γ-tocopherol levels varied significantly (Table 1). EVOOs from Peloponnese displayed the highest concentration of α-tocopherol, which was significantly different (*p* < 0.05) from those of Crete and Lesvos. Moreover, the content of γ-tocopherol was significantly higher in olive oils from Lesvos (northern Aegean Sea).

**Figure 2:**
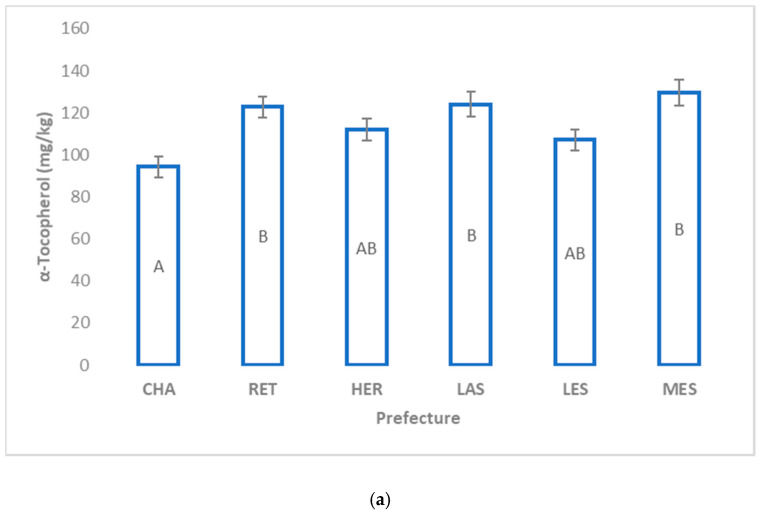

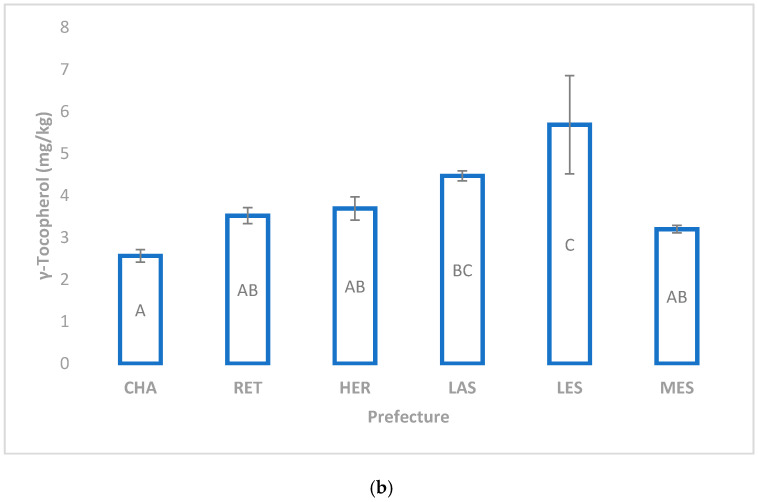
Content (mean ± standard error) of: (**a**) α-tocopherol and (**b**) γ-tocopherol in EVOOs from the Koroneiki cultivar produced in various regions of Greece. Different letters indicate significant difference (Tukey’s HSD test, *p* ≤ 0.05) between the prefectures. CHA: Chania; HER: Heraklion; LAS: Lasithi; RET: Rethymno; LES: Lesvos; MES: Messenia.

When the sub-regions of Crete were examined in combination with the other two areas, Messenia and Lesvos (Figure 2a), the highest mean level of α-tocopherol was observed at Messenia (129 mg/kg) and the lowest was observed at Chania prefecture (94 mg/kg). Interestingly, the α-tocopherol content of EVOO samples from Rethymno (western prefecture of Crete) and Lasithi (eastern prefecture of Crete) did not differ significantly from those of Messenia. Regarding γ-tocopherol (Figure 2b), EVOOs from Lesvos displayed the highest concentration (6 mg/kg) from all the other sub-regions. Lasithi, located at the eastern part of Crete, reserves the highest α- and γ-tocopherol content from the other prefectures of Crete.

### 2.2. Fatty Acid Composition

The main fatty acids in olive oil are oleic (18:1), linoleic (18:2), palmitic (16:0), and stearic acid (18:0). The fatty acid composition is a quality parameter and an authenticity indicator of virgin olive oils [19]. As shown in Table 2, oleic acid displayed the highest content that ranged between 76.60% (Koroneiki from Lesvos) and 71.92% (Kolovi from Lesvos). Linoleic acid ranged between 11.63% (Kolovi from Lesvos) and 5.71% (Koroneiki from Chania), while the corresponding values for palmitic acid ranged between 13.61% (Koroneiki from Chania) and 9.65% (Kolovi from Lesvos). The stearic acid content ranged between 2.76% (Koroneiki from Chania) and 2.20% (Kolovi from Lesvos). The fatty acids, such as palmitoleic (16:1), linolenic (18:3), arachidic (20:0), gadoleic (20:1), and behenic (22:0) acid, were detected in smaller amounts (<0.9%). The content of myristic (14:0), margaric (17:0), heptadecenoic (17:1), and lignoceric (24:0) acid did not exceed 0.1%.

### 2.3. Classification of EVOOs According to Cultivar

**Figure 3:**
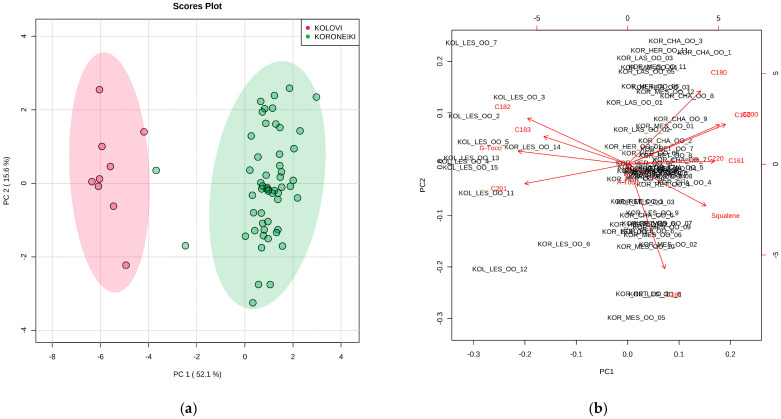
(**a**) Principal Component Analysis (PCA) score plot derived from the concentration of fatty acids, squalene, and α-, γ-tocopherol of EVOOs. The legend indicates the two cultivars tested (Koroneiki, Kolovi) and the colored areas represent the 95% confidence region; (**b**): PCA biplot showing which variables influence the principal components.

The whole dataset (68 EVOOs) were subjected to multivariate statistical analysis in order to investigate if the parameters examined herein (fatty acids, squalene, α- and γ-tocopherol) could discriminate the two olive oil cultivars. Principal Component Analysis (PCA) was carried out, and the results obtained are shown in Figure 3. The fatty acids 14:0, 17:0, 17:1, and 24:0 were excluded from the analysis due to their very low content (<0.1%).

It is evident (Figure 3a) that samples produced from the Koroneiki cultivar form a distinct group (green area), which is clearly separated from that of Kolovi (red area). The two Koroneiki samples that lie outside the confidence eclipse originated from Lesvos. The two principal components explain 67.7% of the total variance. Examining the loading plot (Figure 3b), samples from the Kolovi cultivar are positively correlated with the content of γ-tocopherol as well as linoleic (18:2), linolenic (18:3), and gadoleic (20:1) acid. The remaining fatty acids (16:0, 16:1, 18:0, 18:1, 20:0, and 22:0) and squalene correlated positively with the Koroneiki EVOOs. When student’s *t*-test was applied, it turned out that all variables were statistically significant (*p* < 0.05) between the two cultivars except from α-tocopherol, implying that its content is not dependent on variety. 

**Figure 4:**
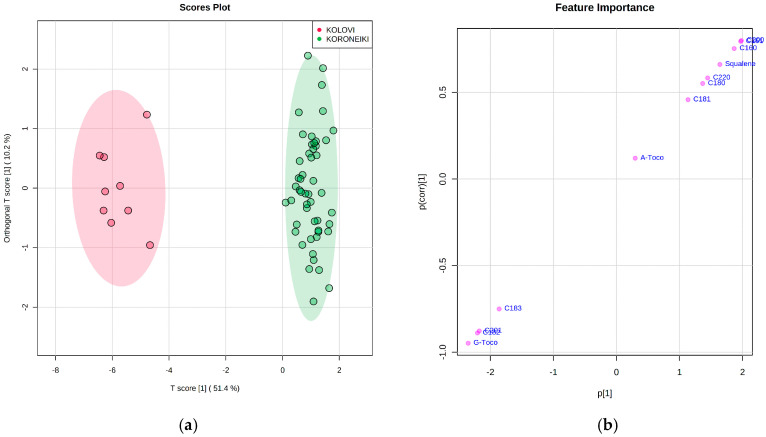
(**a**) Orthogonal Partial Least Squares Discriminant Analysis (OPLS-DA) score plot for the cultivar of EVOOs. The legend indicates the two cultivars tested, and the colored areas represent the 95% confidence region; (**b**) S-plot visualizing the variable influence in the model.

Subsequently, Orthogonal Partial Least Squares Discriminant Analysis (OPLS-DA) was performed in order to find the discriminant features that determine the variety of EVOOs. It is evident that the supervised model can classify the samples into the correct cultivar. Figure 4a illustrates the score plot of OPLS-DA modeling, with a T score [1] of 51.4%, which shows the relevance of the predictive component [1] in explaining the clustering model. Cross-validation (CV) was applied in order to ensure model reliability. The cross-validated predictive ability (Q2) and the explained variation (R^2^(Y)) of the model was 96% (Figure S1). The significance of class discrimination was verified by performing a permutation test (*p* < 0.001; 0/1000) (Figure S2). The variable importance in the OPLS-DA model is shown in Figure 4b. The significant components that contribute to the sample grouping can be identified. Components with a negative p(corr) [1] value display higher levels in the Kolovi cultivar, and on the other hand, features with a positive p(corr) [1] value show higher levels in the Koroneiki cultivar. α-Tocopherol had a low correlation value, which means a low reliability for class separation.

**Figure 5:**
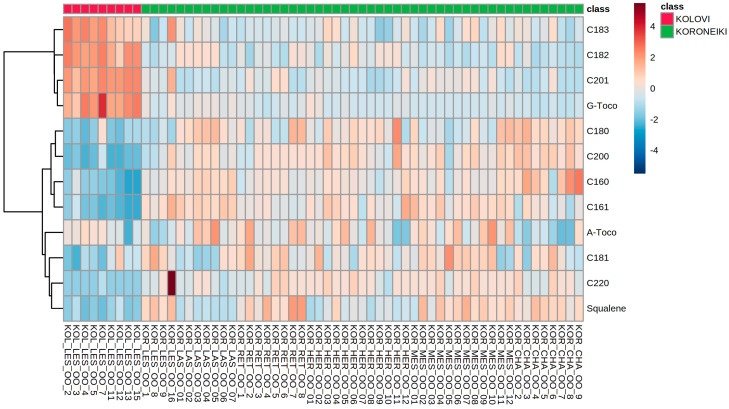
Clustering result of EVOOs by variety in the form of heatmap. The color scale on the right represents the normalized and scaled content of each variable, with red and blue-colored cells indicating high and low content, respectively.

The results can be better visualized using a heatmap (Figure 5). γ-Tocopherol, 20:1, 18:2, and 18:3 are present at higher levels in samples from the Kolovi cultivar and lower in the Koroneiki cultivar. The rest of the components display the opposite pattern, except for α-tocopherol, which does not show a specific pattern between the two cultivars.

## 3. Discussion

### 3.1. Squalene and Tocopherol Content

The squalene content of our samples varied between 4534 and 11,919 mg/kg, which is considered relatively high compared with the literature values. Squalene is known to occur in concentrations between 200 and 7500 mg/kg, although much higher levels (up to 12,000 mg/kg) have been reported [3]. Good-quality Greek olive oils have been found to have average squalene concentration between 2000 and 7000 mg/kg [8,15], and within this range, olive oils from Spain, Italy, and the northern Adriatic region have also been reported [9,31,32].

According to our results, the squalene content of EVOOs was highly influenced by cultivar. Different cultivars from Spain that were grown under the same conditions [10] were reported to have a genetic dependency that explained 96% of the observed squalene variability. Ambra et al. [7] also reported a cultivar-dependent concentration of squalene when they tested EVOOs from Italy. Fernández-Cuesta et al. [33] compared four genotypes, and the results showed large differences between the Spanish cultivars ‘Picual’ and ‘Arbequina’ for squalene content. Kalogeropoulos and Tsimidou in their review paper [15] also stated that the genetic factor can influence the squalene concentration in Greek virgin olive oils. A lot of studies indicate a decreasing formation rate of squalene during the ripening of the olive fruit [22,34] and storage of olive oils [31]. Additionally, the processing technology strongly influences the levels of squalene with a much lower squalene content reported in refined and deodorized olive oils [5]. Although there are some studies indicating the effect of geographical origin on squalene content [35,36], the results of the current study could not support this. 

The α-tocopherol content of the examined EVOOs (73 to 202 mg/kg) is within the range reported for Greek EVOOs. Psomiadou et al. [37] stated that Greek olive oils have α-tocopherol levels that are among the highest found, ranging from 98 to 370 mg/kg, although even higher levels have been reported for olive oils from Tunisia (478 mg/kg) [34] and Spain (502 mg/kg) [23]. Relatively high concentrations have also been found for the Koroneiki cultivar from different regions [38,39] ranging from 324 to 350 mg/kg. According to the literature [13], the content of γ-tocopherol ranges between 8.9 and 13.4 mg/kg; these values that are in good agreement with the results of the present study (2 to 20 mg/kg). Béltran et al. [23], studying different cultivars from Spain, concluded that the cultivar was the most important variation factor for γ-tocopherol. This is in agreement with our results. It should be mentioned here that the olive oil-producing season of 2018–2019 in Greece was characterized by a reduced production due to biennial bearing effects of olive trees as well as the development of fungal diseases due to unfavorable weather conditions. Thus, the content of tocopherols was probably affected negatively, considering that the olive growers might have applied more intensive extraction conditions in order to increase olive oil production.

There are several studies that have described α-tocopherol as cultivar dependent [7,38,40,41]; however, this was not supported by our results. Besides the genetic factor, agronomic (climate, crop year, ripening of the olive fruit, irrigation, rainfall, and soil quality) and technological factors (processing and storage) have been reported to affect tocopherol concentration [15]. As far as the geographical factor is concerned, Bogres et al. [24] studied samples of the variety “Arbequina” originated from different areas of Spain and Brazil. They concluded that climatic conditions, and therefore the region of origin, significantly affect tocol concentration. Another study conducted in Tunisia by Dabbou et al. [42] managed to differentiate olive oils from the same cultivar grown in different areas based on their α-tocopherol content. 

### 3.2. Fatty Acid Composition

The mean values of fatty acids found in the present study were within the limits established by the IOOC (International Olive Oil Council) for purity criteria of olive oils [43]. The results of our research indicate that the application of chemometric methods to squalene, tocols, and fatty acid composition can be quite successful for the classification of EVOOs with respect to cultivar. Similarly, Kosma et al. [14], while investigating the differentiation of Greek EVOOs, concluded that variations in fatty acid composition may be owed primarily to factors such as cultivar and secondarily to climatic conditions and geographical origin. Similar results emerged from Stefanoudaki et al. [18], who studied the cultivars Koroneiki and Mastoides from Crete, but they also posited the possibility of altitude and rainfall having an impact. Diraman et al. [19] achieved the classification of different Turkish olive oils based on the variety, geographical origin, and harvest year, using their fatty acid composition. Subsequently, a large effort has been made to study the effects of geographical origin on fatty acid content. According to Tsimidou and Karakostas [17], the non-parametric discriminant analysis of fatty acid compositional data may prove to be valuable for the classification of virgin olive oils according to their origin. Finally, Youssef et al. [44] studied olive oil samples of the Questati cultivar collected from seven different areas in Tunisia and reported a greater variation in the palmitic, linoleic, and oleic acid content. They concluded that a possible cause of this variation was the interaction of variety with environmental conditions during the growth and maturation of olive fruits.

## 4. Materials and Methods 

### 4.1. Olive Oil Samples

A total of 68 monovarietal EVOOs were obtained during the harvesting period of 2018–2019 (Table 3). They were produced from two cultivars (Kolovi and Koroneiki) in three different geographical regions of Greece (Crete island, Lesvos island, and Peloponnese). The samples were packaged in dark-brown glass bottles and stored at 4 °C until analysis. 

### 4.2. Determination of Fatty Acids and Squalene 

The fatty acid composition was determined according to the official method of the Commission Regulation (EEC) No 2568/91, which was slightly modified for the co-determination of squalene [45]. Briefly, the fatty acid methyl esters were prepared by vigorous shaking of an olive oil solution in heptane, containing tetradecane (200 mg/L) (99%, ACROS Organics, Geel, Belgium) as an internal standard, with 0.2 mL of 2 M methanolic potassium hydroxide in screwcap vials. The mixture was left to stratify until the upper phase became clear and an aliquot was taken for gas chromatographic analysis. Analytical grade methanol, heptane, and potassium hydroxide were purchased from Merck (Darmstadt, Germany). 

The samples were analyzed on a Shimadzu GC-2010 Plus chromatograph (Shimadzu Corporation, Kyoto, Japan) equipped with a flame ionization detector and autosampler. The injection volume was 1 μL in split mode (split ratio 1/50). The separation of fatty acid methyl esters and squalene was accomplished in a CP Wax 52CB capillary column (30 m × 0.32 mm i.d., × 0.25 μm film thickness, Agilent, Santa Clara, CA, USA). The carrier gas was helium at a constant linear velocity of 28 cm/s. The temperature of the injection port and the detector were set at 250 and 270 °C, respectively. The oven temperature was maintained initially at 100 °C for 1 min; then, it was programmed at 25 °C/min to 200 °C and held for 1 min. Then, it increased to 230 °C with a rate of 3 °C/min and remained there for 6 min. Finally, the oven temperature increased to 250 °C with a rate of 30 °C/min and was held constant for 5 min. Peak identification was effected using reference compounds (Supelco 37 Component FAME mix, Sigma Aldrich, Darmstadt, Germany). The content of each fatty acid was expressed as percentage m/m using the peak area. Squalene content (expressed as mg/kg) was calculated from its corresponding calibration curve using the internal standard method. 

### 4.3. Determination of Tocopherol Content

The determination of α-, β-, γ-, and δ-tocopherols and tocotrienols was performed according to ISO 9936 (2006), using high-performance liquid chromatography with fluorescence detection [46]. In brief, a JASCO HPLC system (JASCO International Co., Ltd., Tokyo, Japan) was used, consisting of a quaternary pump (PU-2089 Plus), an autosampler (AS-1555), and a fluorescence detector (FP-920). Separation was accomplished with a Pinnacle DB Silica column (250 mm × 4.6 mm i.d., 5 μm, Restek, USA) using isocratic elution with *n*-Hexane/1,4-Dioxane (97:3 *v/v*). The flow rate was set at 1.5 mL/min, and the injection volume was 20 μL. Analytical grade hexane was purchased from Merck and 1,4-dioxane was purchased from LAB-SCAN (Labscan International Ltd., Dublin, Ireland). The excitation and emission wavelength were set at 295 nm and 330 nm, respectively. The content of each tocol was calculated using the calibration factor of a standard solution of (±)-α-tocopherol (Merck, Darmstadt, Germany) and expressed in mg/kg.

### 4.4. Statistical Analysis

Analysis of variance was applied to the squalene and tocopherols data using STATISTICA software (version 10.0, StatSoft Inc., Tulsa, OK, USA). Tukey’s test was applied to check differences of means at *p* = 0.05. Multivariate data analysis was accomplished using the web-based tool suite MetaboAnalyst 4.0 [47]. Before statistical processing, the data were auto-scaled in order to remove the overall offset [48].

## 5. Conclusions

In the present study, 68 EVOOs originated from the three major olive oil-producing areas of Greece—Crete island, Lesvos island and Peloponnese—belonging to two local cultivars, Kolovi and Koroneiki, were studied for their squalene and tocopherols content as well as fatty acid composition. The squalene content of olive oils from the Koroneiki cultivar was significantly higher than that of the Kolovi cultivar. The influence of the geographical origin was not so clear, considering that while the samples differed significantly between the eastern (Lassithi, Heraklion) and western (Chania, Rethymno) part of Crete, this was not the case for the rest of the regions tested (Messenia, Lesvos). It is also remarkable that all the olive oils studied, regardless of cultivar and region of origin, were characterized by relatively high squalene content compared to the values mentioned in the literature. As regards tocols, the cultivar did not appear to have a significant effect on α-tocopherol content, in contrast to the findings of other researchers. On the other hand, γ-tocopherol differed significantly between the two cultivars. As far as the geographical origin is concerned, the α-tocopherol content of samples from Peloponnese was significantly higher than that of samples from Crete and Lesvos, whereas γ-tocopherol was present in higher concentrations in EVOOs from Lesvos. As regards fatty acids, the Kolovi cultivar was positively correlated with the content of linoleic (18:2), linolenic (18:3), and gadoleic (20:1) acid, whereas the Koroneiki cultivar was positively correlated with the rest of the fatty acids determined and squalene. The results of the present study show that the application of multivariate analysis on the compositional data of EVOOs is a promising tool for their discrimination based on geographical origin and/or cultivar. However, due to the complexity of olive oil composition as influenced by numerous factors, further research should be conducted that should include samples from subsequent harvesting cycles, in order to increase the discriminating power of statistical techniques. We believe that the current study will help to highlight the unique characteristics of Greek olive oils, which will benefit both consumers and producers.

## Figures and Tables

**Table 1:** Content ^1^ of squalene and tocopherols (expressed as mg/kg, mean ± standard deviation) in monovarietal extra virgin olive oils from different regions of Greece.

Factor ^2^	Squalene	α-Tocopherol	γ-Tocopherol
*Variety*			
KOR	8576 ± 1546 ^A^	117 ± 21 ^A^	4 ± 1 ^A^
KOL	5440 ± 822 ^B^	110 ± 15 ^A^	13 ± 3 ^B^
*Region*			
CRE	8223 ± 1569 ^A^	112 ± 19 ^A^	4 ± 1 ^A^
LES	8901 ± 1660 ^A^	107 ± 12 ^A^	6 ± 3 ^B^
PEL	9207 ± 1295 ^A^	130 ± 23 ^B^	3 ± 0 ^A^

^1^ Different letters next to the columns (^A^, ^B^) indicate significant differences (Tukey’s HSD test, *p* ≤ 0.05) within each factor; ^2^ KOR: Koroneiki; KOL: Kolovi; CRE: Crete; LES: Lesvos; PEL: Peloponnese. Samples from different regions were produced from the Koroneiki cultivar.

**Table 2:** Fatty acid composition (% m/m methyl esters) of Greek EVOOs by variety and region.

Variety Region ^1^	% m/m	14:0	16:0	16:1	17:0	17:1	18:0	18:1	18:2	18:3	20:0	20:1	22:0	24:0
Kolovi														
LES	mean	0.01	9.65	0.55	0.05	0.08	2.20	74.06	11.63	0.84	0.37	0.38	0.12	0.06
	SD ^2^	0.00	0.88	0.07	0.01	0.01	0.19	1.64	1.06	0.06	0.02	0.01	0.00	0.01
Koroneiki														
LES	mean	0.01	11.27	0.78	0.04	0.08	2.30	76.60	7.22	0.74	0.42	0.33	0.16	0.06
	SD	0.01	0.97	0.11	0.01	0.01	0.10	1.33	1.81	0.12	0.03	0.04	0.04	0.01
MES	mean	0.01	11.96	0.83	0.04	0.07	2.53	76.47	6.45	0.69	0.45	0.30	0.15	0.06
	SD	0.00	0.52	0.05	0.00	0.00	0.21	1.71	1.07	0.06	0.02	0.01	0.01	0.00
CHA	mean	0.01	13.61	0.73	0.04	0.07	2.76	75.46	5.71	0.67	0.47	0.28	0.15	0.06
	SD	0.00	1.72	0.07	0.00	0.01	0.12	1.60	0.67	0.05	0.01	0.02	0.01	0.00
HER	mean	0.01	11.84	0.79	0.04	0.07	2.64	75.63	7.38	0.67	0.45	0.28	0.15	0.05
	SD	0.00	0.53	0.09	0.00	0.00	0.22	0.86	0.80	0.06	0.02	0.02	0.01	0.01
LAS	mean	0.01	12.53	0.86	0.04	0.08	2.72	74.59	7.57	0.68	0.45	0.28	0.14	0.05
	SD	0.00	0.38	0.03	0.01	0.02	0.13	1.29	1.25	0.04	0.01	0.01	0.01	0.00
RET	mean	0.01	11.77	0.75	0.04	0.07	2.57	76.58	6.60	0.67	0.44	0.29	0.15	0.06
	SD	0.00	0.40	0.03	0.00	0.01	0.24	1.20	1.12	0.02	0.01	0.02	0.01	0.01

^1^ LES: Lesvos; MES: Messenia; CHA: Chania; HER: Heraklion; LAS: Lasithi; RET: Rethymno ^2^ SD: standard deviation.

**Table 3:** Number and coding of Greek olive oil samples according to cultivar and geographical origin.

	Region/Prefecture ^1^					
Variety	LES	PEL	CRE			
		*MES*	*CHA*	*RET*	*HER*	*LAS*
Kolovi	9	-	-	-	-	-
Koroneiki	6	17	9	8	12	7

^1^ LES (Lesvos), PEL (Peloponnese), MES (Messenia), CRE (Crete), CHA (Chania), RET (Rethymno), HER (Heraklion), LAS (Lasithi).

## Supplementary Materials

The following are available online, Figure S1: OPLS-DA model overview, Figure S2: Permutation test of OPLS-DA model.
