# Supplementary figures and images for: Varietal and Geographical Discrimination of Greek Monovarietal Extra Virgin Olive Oils Based on Squalene, Tocopherol, and Fatty Acid Composition
Source: Molecules. 2020 Aug 21;25(17):3818. doi: 10.3390/molecules25173818 (PMC7503666; doi:10.3390/molecules25173818)

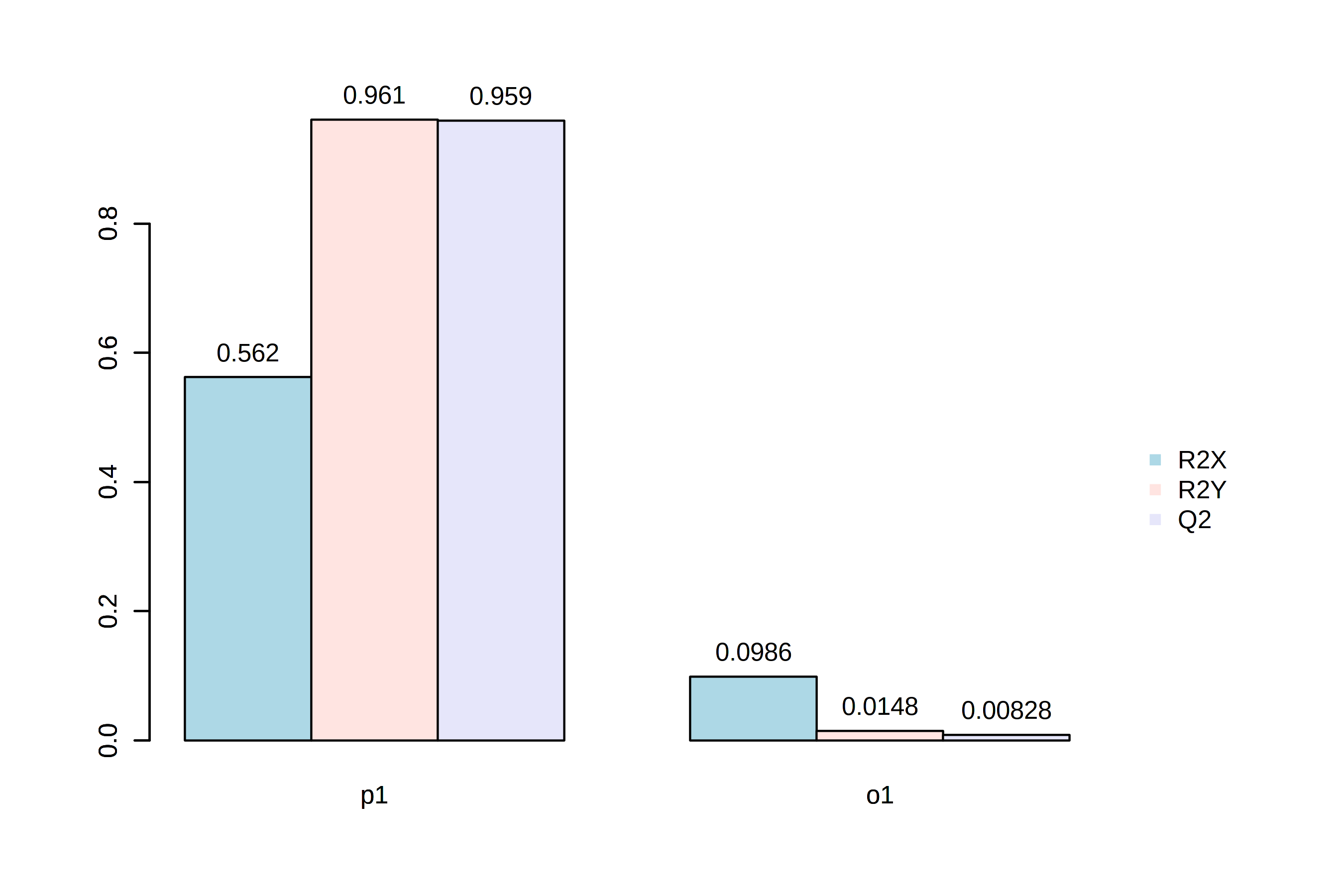

Supplement: Supplementary file 1 [file molecules-25-03818-s001.zip › Figure S1.tiff]

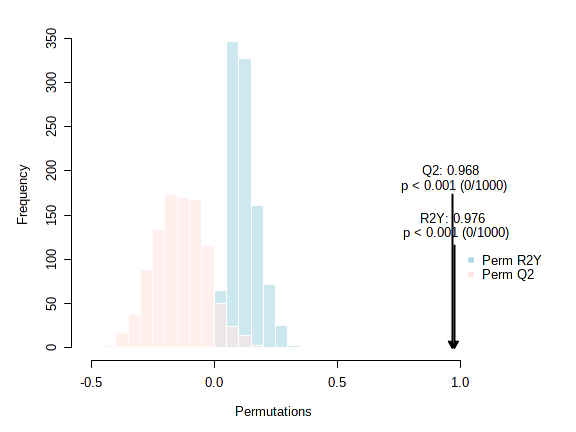

Supplement: Supplementary file 1 [file molecules-25-03818-s001.zip › Figure S2.png]
